# Screening and experimental validation of modified Gandou Decoction-targeted inhibitors for alleviating AD components via network pharmacology, machine learning, and molecular dynamics simulation

**DOI:** 10.3389/fphar.2025.1685866

**Published:** 2025-10-31

**Authors:** Shixin Ye, Shun Zhang, Liangdong Zhang, Guorong Peng, Ming Xie, Xiongfeng Huang, Yousheng Hu

**Affiliations:** ^1^ Fuzhou Medical College, Nanchang University, Fuzhou, Jiangxi, China; ^2^ Fuzhou Medical University, Fuzhou, Jiangxi, China; ^3^ Teaching and Research Section of Anatomy, Basic Medical College, Fuzhou Medical College, Nanchang University, Fuzhou, Jiangxi, China; ^4^ Department of Postgraduate, Jiangxi University of Chinese Medicine, Nanchang, Jiangxi, China; ^5^ Fuzhou Key Laboratory of Chronic Disease Research, Fuzhou Medical College, Nanchang University, Fuzhou, Jiangxi, China

**Keywords:** Alzheimer’s disease, molecular targets, mechanisms, network pharmacology, molecular docking, molecular dynamics simulation, experimental validation

## Abstract

**Background:**

Alzheimer’s disease (AD) is a neurodegenerative disease characterized by abnormal accumulation of β-amyloid (Aβ) and hyperphosphorylation of the Tau protein. Currently, there is a lack of effective and safe therapeutic approaches. In Traditional Chinese medicine (TCM), Gandou Decoction has shown significant efficacy in improving cognitive decline and dementia-related symptoms, but its specific mechanism remains unclear.

**Methods:**

This study systematically analyzed the active components and anti-AD mechanism of Modified Gandou Decoction (MGD) by integrating network pharmacology, machine learning, molecular docking, molecular dynamics (MD) simulation, and *in vitro* experimental validation. Obtain the components of Chinese medicines in MGD from TCMSP and screen them via ADMET; obtain AD targets by combining databases and select core targets through machine learning; verify their effects through various analyses and experiments.

**Results:**

A total of 21 potential active molecules of MGD and 68 intersection targets were screened out. Among them, 8 core targets (EIF2AK2, PPARG, BACE1, ESR1, GSK3B, ACE, CASP3, MAPK14) were confirmed to be significantly associated with AD pathology by gene expression difference analysis (P ≤ 0.05). KEGG enrichment analysis showed that MGD mainly intervenes in the amyloid production pathway, the MAPK pathway, and the IL-17 pathway. Molecular docking demonstrated that the majority of the 21 potential active compounds exhibited strong binding affinities to the 8 core targets. Moreover, some potential active molecules exhibited better binding energy and similar binding modes compared with known inhibitors when binding to the corresponding target proteins. Molecular dynamics simulation showed that Alisol B, a potential active component of MGD, could stably bind to BACE1, EIF2AK2, and CASP3. *In vitro* cell experiments confirmed that Alisol B could significantly reverse okadaic acid-induced damage in SH-SY5Y cells (p < 0.001).

**Conclusion:**

MGD exerts its anti-AD effect through its potential active component Alisol B, which binds to target proteins BACE1, EIF2AK2, and CASP3, and synergistically inhibits Aβ production, Tau phosphorylation, and neuroinflammatory processes through multiple pathways. This study provides a foundation for developing MGD-derived natural products for AD treatment, although the precise mechanisms require further experimental validation.

## Graphical Abstract

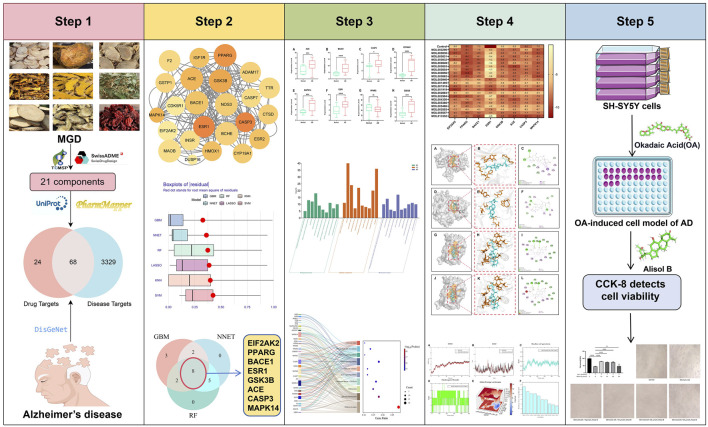

## 1 Introduction

Alzheimer’s disease (AD), a progressive neurodegenerative disorder and the leading cause of dementia globally, constitutes a significant global health burden affecting an estimated 40 million people, with prevalence projected to rise in the coming decades (Breijyeh and Karaman, 2020; Twarowski and Herbet, 2023) Its primary clinical manifestations involve progressive memory loss and cognitive impairment, which severely compromise patients’ ability to perform basic activities of daily living (Thakral et al., 2023). As the disease progresses, patients eventually die from complications such as infection, dysphagia, or malnutrition (Pang et al., 2018; Scheltens et al., 2021). The main pathogenesis of AD involves: inflammatory reactions caused by excessive accumulation of free β-amyloid (Aβ) forming Aβ plaques, and neurodegeneration of neurons due to neurofibrillary tangles caused by microtubule-associated (Tau) protein denaturation (Kapasi et al., 2021). Currently, there are no specific drugs for AD treatment; FDA-approved drugs can only improve cognitive function in the short term and are accompanied by significant adverse reactions (Wong et al., 2022). With the acceleration of global population aging, the demand for safe and effective new drugs has become increasingly urgent.

Traditional Chinese medicine (TCM) emphasizes syndrome differentiation and holistic treatment, often achieving unique efficacy, especially for complex diseases such as neurodegenerative diseases, cancer, and diabetes (Gao et al., 2014; Cai et al., 2025). Since 1970, the Institute of Neurology at Anhui University of Chinese Medicine has utilized Gandou Decoction (GDD), which possesses effects of clearing heat, detoxifying, promoting bowel movement, and draining dampness, to treat Wilson’s disease (WD) patients, achieving good clinical efficacy (Xue et al., 2007). Modified Gandou Decoction (MGD) is derived from the original GDD with the addition of several herbal components known to promote blood circulation and nourish the marrow. MGD consists of *Rhei Radix ET Rhizoma* (Dahuang), *Coptidis Rhizoma* (Huanglian), *Curcumae Rhizoma* (Ezhu), *Curcumae Longae Rhizoma* (Jianghuang), *Houttuyniae Herba* (Yuxingcao), *Alismatis Rhizoma* (Zexie), *Notoginseng Radix ET Rhizoma* (Sanqi), *Paeoniae Radix Alba* (Baishao), and *Corni Fructus* (Shanzhuyu). Researchers demonstrated that MGD ameliorates copper overload-induced neuronal damage by downregulating the expression levels of ASM, Cer, and p38 MAPK at both mRNA and protein levels in the ceramide signaling pathway in Wilson’s disease model mice brain tissue (Xu et al., 2017b). In another study by the same research group, it was further revealed that MGD facilitates the elimination of excess copper and suppresses the expression of cytochrome C (Cyt C), Caspase-9, and caspase-3 in neurons, thereby modulating the Cyt C/caspase signaling pathway. These mechanisms contribute to the amelioration of brain injury and support the recovery of cognitive function in patients with Wilson’s disease (Xu et al., 2017a). MGD has been confirmed to regulate the expression of synaptic-related proteins, alleviate synaptic dysfunction, and further improve brain injury (Hu et al., 2004; Feng et al., 2025). Therefore, it is anticipated that by adjusting the composition of MGD, its components that alleviate cognitive decline can be retained for AD treatment.

Network pharmacology analyzes the interaction mechanism between drugs and diseases from a holistic perspective by constructing a “drug-component-target-pathway-disease” network (Nogales et al., 2022). Combined with machine learning, it can effectively screen large and complex datasets, predict drug molecules with therapeutic potential through computational simulation, and provide effective methods and scientific basis for analyzing the therapeutic mechanism of TCM, as well as explain its active components and mechanism of action at the molecular level (Yang et al., 2020).

This study analyzed the active components, targets, and pathways of MGD in AD treatment using network pharmacology, machine learning, molecular docking, molecular dynamics simulation, and other techniques, and further verified through cell experiments. This research can promote the study of Chinese medicine prescriptions for AD treatment and accumulate useful knowledge for the development of natural drugs for AD.

## 2 Materials and methods

### 2.1 Acquisition of potential active components of MGD

Compounds of the 9 Chinese herbs in MGD were retrieved from the TCMSP database (https://www.tcmsp-e.com/), and the properties of these compounds were predicted using admetSAR and SwissADME databases. Potential active components of MGD for AD treatment were screened according to the criteria: OB ≥ 30%, DL ≥ 0.18, compliance with Lipinski’s five rules, blood-brain barrier permeability, and no hepatotoxicity.

### 2.2 Prediction of potential targets of MGD against AD

The 3D chemical structures of MGD’s potential active components were retrieved from the PubChem database and input into the PharmMapper database for target prediction; targets with a Norm fit score ≥0.9 were considered highly relevant. Standardization was performed using the UniProt database, and relevant literature was retrieved for supplementation. Using “Alzheimer’s disease” as the keyword, AD-related targets and their scores were retrieved from the DisGeNet database. Drug targets and disease targets were imported into Venny 2.1.0 to generate a Venn diagram. The intersection targets in the Venn diagram were the potential targets of MGD against AD.

### 2.3 Construction of protein-protein interaction (PPI) network

To further study the interactions between MGD’s potential anti-AD targets, they were imported into the STRING database (https://cn.string-db.org/) with the standard set to “*Homo sapiens*” and the minimum interaction score >0.4 (Li et al., 2024). The PPI network was visualized using Cytoscape 3.9.1. Important protein subnetworks were screened based on disease relevance scores from the DisGeNet database.

### 2.4 Screening of core targets of MGD for AD treatment using machine learning algorithms

AD gene expression profiles were retrieved from the Gene Expression Omnibus (GEO) database (https://www.ncbi.nlm.nih.gov/geo/). Expression of key genes in the hippocampus of 60 samples (47 AD samples and 13 normal samples) was obtained from GSE5281, GSE9770, and GSE28146. To further screen the core genes of MGD against AD, six machine learning algorithms were used: Gradient Boosting Machine (GBM), Neural Network (NNET), Random Forest (RF), Least Absolute Shrinkage and Selection Operator (LASSO), K-Nearest Neighbor (KNN), and Support Vector Machine (SVM). Each model was then tested using the DALEX package to obtain cumulative residual distributions and box plots, and the area under the receiver operating characteristic curve (AUC) was used to evaluate the fitting degree of each model (Xiong et al., 2023).

### 2.5 Differential expression analysis of core genes

The expression levels of the 8 core genes in the hippocampus were obtained from 60 samples in the GSE5281, GSE9770, and GSE28146 datasets. R packages were used to analyze the differential expression of core genes in the hippocampus between AD patients and healthy controls. GraphPad Prism 10 was used for statistical analysis and box plot visualization, with t-tests (and non-parametric tests) for statistical significance. Data are expressed as mean ± SD. *: p ≤ 0.05; **: p ≤ 0.01; ***: p ≤ 0.001; ****: p ≤ 0.0001. This can verify the consistency of core genes screened by machine learning algorithms; the mechanism of MGD against AD was further analyzed by evaluating the upregulation or downregulation trends of core gene expression.

### 2.6 GO and KEGG enrichment analysis

The Database for Annotation, Visualization and Integrated Discovery (DAVID) (https://david.ncifcrf.gov) was used for Gene Ontology (GO) enrichment analysis and Kyoto Encyclopedia of Genes and Genomes (KEGG) pathway enrichment analysis of intersection genes (Zheng et al., 2023). In addition, the top 10 GO terms and KEGG pathways with the lowest p-values were visualized using an online platform (http://www.bioinformatics.com.cn/). Key targets and core targets were mapped to key pathways using KEGG mapper (Zhou et al., 2024).

### 2.7 Construction of drug-component-target-pathway-disease network

To study the complex regulatory network of MGD on AD, the potential active components, targets, and related pathways of MGD were input into Cytoscape 3.9.1 to establish a drug-component-target-pathway-disease network.

### 2.8 Molecular docking

Semi-flexible docking was used in this study. The structures of MGD’s potential active components were obtained from the TCMSP database (Chen and Seukep, 2020). 3D models of Core target protein were obtained from the Protein Data Bank database (Berman and Burley, 2025) (PDB) (https://www.rcsb.org/): 1GFW (CASP3) (Lee et al., 2000), 7MSA (ESR1) (Liang and Zbieg, 2021), 1Q3W (GSK3B) (Iwaloye et al., 2020), 1UZF (ACE) (Natesh et al., 2004), 2RG6 (MAPK14) (Hynes et al., 2008), 2OHP (BACE1) (Hernández-Rodríguez et al., 2016), 6D3K (EIF2AK2) (Mayo et al., 2019),7AWD(PPARG) (Willems et al., 2021). These 3D structures were subjected to preprocessing, including the removal of all water molecules and the addition of hydrogen atoms (Moharana and Pattanayak, 2023).

The 3D chemical structures of MGD’s potential active components were input into AutodockTools 1.5.7, with hydrogen atoms and charges added. Docking box sites were then defined according to the position of positive control molecules, and semi-flexible docking was performed using Autodock Vina to calculate binding energy. Positive control molecules were known inhibitors of core target proteins recorded in the database. Finally, PyMOL(v.2.5.7) and Discovery Studio Visualizer were used to visualize the interactions between proteins and MGD’s potential active molecules, and their binding poses and binding interactions were analyzed.

### 2.9 Molecular dynamics simulation

After molecular docking, Gromacs 2020.6 was used for molecular dynamics (MD) simulation to further analyze the stability of complexes formed by potential active molecules with good binding (low binding energy and high similarity in binding interactions to positive controls) and target proteins. Potential active components of MGD were preprocessed on the ACPYPE server website (https://www.bio2byte.be/acpype/submit/). MD simulation conditions included a constant temperature of 300K and atmospheric pressure of 1Bar; the simulation system used the Amber99sb-ildn force field, with water as the solvent, and Cl^−^ and Na^+^ were added to stabilize the electroneutral system (Shi et al., 2023). Energy minimization was performed using the steepest descent method. Subsequently, equilibration was performed for 100,000 steps under NVT and NPT ensembles. Finally, an MD simulation was conducted.

Qtgrace and DuIvyTools were used to visualize MD simulation results, including root mean square deviation (RMSD), root mean square fluctuation (RMSF), and number of hydrogen bonds (H-bonds). Gibbs free energy (GFE) was calculated using the built-in “g_sham” and “xpm2txt.py” scripts in Gromacs 2020.6 software. Free energy landscapes were plotted based on RMSD and radius of gyration (Rg), and MM/PBSA binding free energy was calculated (Spiliotopoulos et al., 2012).

### 2.10 Cell culture and CCK-8 assay for cell viability

Human neuroblastoma cells (SH-SY5Y) were cultured in DMEM/F12 medium containing 10% fetal bovine serum (FBS) and 1% penicillin-streptomycin at 37 °C with 5% CO_2_.

Logarithmic phase SH-SY5Y cells were uniformly seeded into 96-well plates and incubated for 24 h. Cells were treated with 50 nmol/L okadaic acid (OA) for 6 h to construct an AD cell model (Chen et al., 2016). SH-SY5Y cells were divided into a control group, OA group, and OA + Alisol B group. Cell viability in each group was detected according to the CCK-8 kit instructions (Lu et al., 2021).

### 2.11 Statistical analysis

Statistical analysis was performed using GraphPad Prism 10 software. One-way analysis of variance (ANOVA) and Student-Newman-Keuls (SNK) method were used to evaluate statistical differences. Data are expressed as mean ± SD.

## 3 Results

### 3.1 Acquisition and preliminary screening of potential active components of MGD

According to OB, DL, Lipinski’s five rules, and ADMET screening criteria, 21 potential active components of Modified Gandou Decoction (MGD) were screened from the TCMSP database and related literature. Among them, MOL000296 and MOL000940 were derived from *Curcuma zedoaria*; MOL000830, MOL000831, MOL000832, MOL000849, MOL000853, MOL000854, MOL000856, and MOL000862 from *Alisma orientale*; MOL001495, MOL002883, MOL005360, MOL005486, MOL005503, and MOL005557 from *Cornus officinalis*; MOL001918 and MOL001919 from *Paeonia lactiflora*; MOL002904 and MOL013352 from *Coptis chinensis*; MOL004350 from *Houttuynia cordata*; and none from *Rhubarb*, *Curcuma longa*, or *Panax notoginseng* (Supplementary Table S1).

### 3.2 Acquisition of potential targets of MGD against AD

**FIGURE 1:**
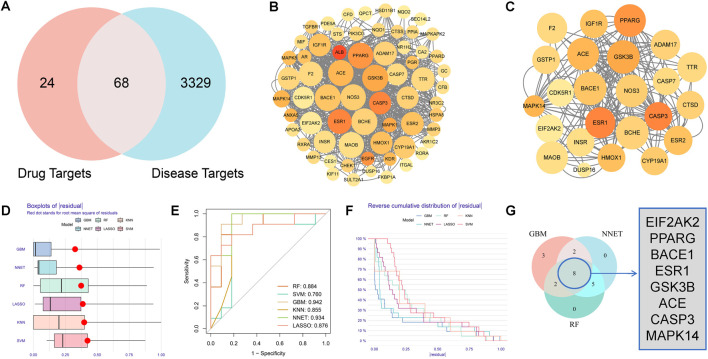
Acquisition of potential targets and machine learning screening of core targets of Modified Gandou Decoction against AD. **(A)** Venn diagram of targets of potential active molecules of Modified Gandou Decoction in the human body and AD disease targets. **(B)** PPI network of 68 potential targets. **(C)** Key subnetwork consisting of 21 targets. **(D)** Residual box plot of AD samples. **(E)** ROC curves of six machine learning models. **(F)** Cumulative residual distribution plot of AD samples. **(G)** Core targets obtained by intersection analysis.

After screening and removing duplicate targets of each Chinese herb component in MGD, 92 targets highly related to MGD’s potential active components were obtained through PharmMapper. 3397 AD-related targets were retrieved from the DisGeNet database. Using Venny 2.1.0, 68 potential targets of MGD against AD were obtained from the intersection (Figure 1A).

### 3.3 Construction of PPI network

To study the interactions between MGD’s anti-AD intersection proteins, a PPI network was constructed using STRING 12.0 and Cytoscape 3.9.1. The network consisted of 65 nodes and 734 edges; each node represents a target protein, with redder nodes indicating higher degree values and more interactions with other target proteins. Larger nodes indicate stronger relevance to the disease (Figure 1B). A key subnetwork was constructed by screening target proteins from cytoHubba with a disease relevance score >0.1, containing 23 nodes and 87 edges (Figure 1C).

### 3.4 Screening of core targets of MGD for AD treatment using machine learning

To further screen the core targets of MGD against AD, this study systematically constructed six machine learning models: Gradient Boosting Machine (GBM), Neural Network (NNET), Random Forest (RF), Least Absolute Shrinkage and Selection Operator (LASSO), K-Nearest Neighbor (KNN), and Support Vector Machine (SVM). Model performance was analyzed using the “DALEX” R package, and residual box plots were drawn. The residual box plot in Figure 1D shows the dispersion of prediction errors of each model; the box length and outlier distribution indicate that GBM and NNET models have more concentrated residual distributions, while KNN and SVM models show larger prediction fluctuations (Figure 1D). The area under the curve (AUC) in Figure 1E was used to quantitatively evaluate model classification performance, and Gradient Boosting Machine (GBM, AUC = 0.942), Neural Network (NNET, AUC = 0.934), and Random Forest (RF, AUC = 0.884) were selected as the optimal models. The residual reverse cumulative distribution plot in Figure 1F further verifies model robustness. The Venn diagram in Figure 1G shows the intersection of the top 15 feature genes from the three models(GBM, NNET, RF), among which 8 genes (EIF2AK2, PPARG, BACE1, ESR1, GSK3B, ACE, CASP3, and MAPK14) were highly weighted in all three algorithms, suggesting that these genes may be key target proteins for MGD in intervening AD.

Among these 8 core proteins, EIF2AK2 is mainly involved in regulating protein translation and inflammatory factor expression (Feng et al., 2025); BACE1 is closely related to Aβ production (Kapasi et al., 2021); GSK3B is involved in Aβ production and Tau protein phosphorylation through metabolic regulation (Lauretti et al., 2020); CASP3 is related to cell apoptosis (Pandya et al., 2025); MAPK14 participates in cellular inflammatory signaling pathways (Prabha et al., 2025).

### 3.5 Differential expression analysis of core protein genes

R packages were used to analyze the differential expression of the 8 core genes screened by machine learning. The expression levels of core genes in the hippocampus of AD patients were compared with those in the normal group (healthy controls). Compared with healthy controls, 7 core genes in the hippocampus of AD patients (EIF2AK2, BACE1, ESR1, GSK3B, ACE, CASP3, and MAPK14) were significantly upregulated, with statistically significant differences (p < 0.05), except for PPARG (Supplementary Figure S1).

### 3.6 GO and KEGG enrichment analysis

**FIGURE 2:**
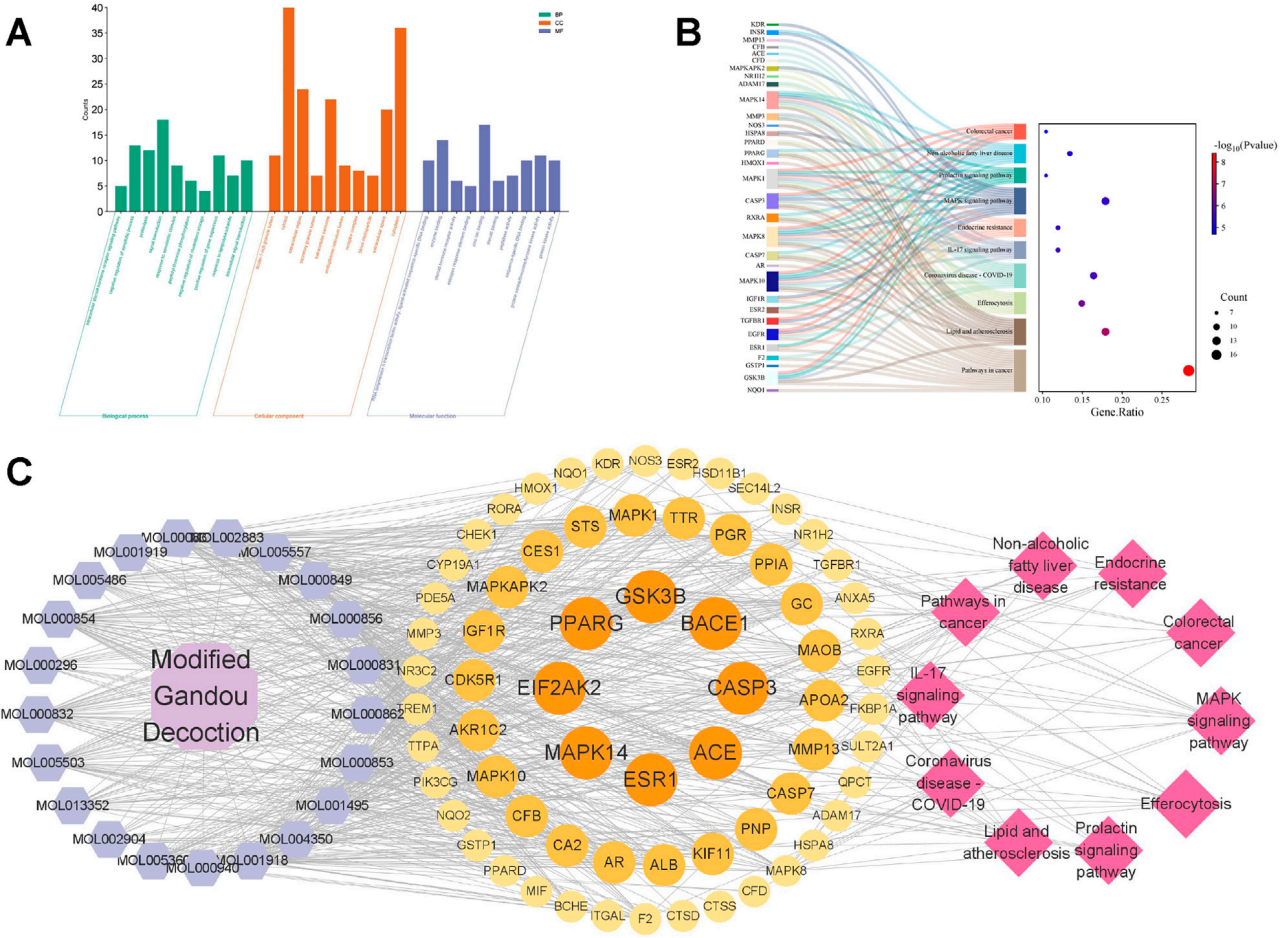
GO and KEGG enrichment analysis. **(A)** The top 10 biological process (BP) terms, cellular component (CC) terms, and molecular function (MF) terms from GO enrichment analysis are represented by green, orange, and purple bars, respectively. **(B)** Sankey diagram of KEGG pathway analysis of potential targets of MGD for AD treatment. Left rectangular nodes represent therapeutic targets, right rectangular nodes represent KEGG pathways, and lines represent the association between targets and pathways. **(C)** Drug-component-target-pathway interaction network. Purple hexagons represent potential active components of MGD, circles represent potential targets, and pink diamonds represent pathways.

A total of 400 GO terms were obtained through GO enrichment analysis, including 300 biological process (BP) terms, 36 cellular component (CC) terms, and 64 molecular function (MF) terms. Figure 2A shows the top 10 GO biological terms. BPs include: intercellular steroid hormone receptor signaling pathway, negative regulation of apoptotic process, proteolysis, signaling transduction, response to xenobiotic stimulus, peptidyl-threonine phosphorylation, negative regulation of cholesterol storage, positive regulation of gene expression, and response to lipopolysaccharide. CCs include: ficolin-1-rich granule lumen, cytosol, extracellular region, secretory granule lumen, extracellular exosome, endoplasmic reticulum lumen, receptor complex, blood microparticle, extracellular space, and cytoplasm. MFs include: RNA polymerase II transcription factor activity, ligand-activated sequence-specific DNA binding, enzyme binding, steroid hormone receptor activity, estrogen response element binding, zinc ion binding, steroid binding, peptidase activity, sequence-specific DNA binding, protein serine/threonine/tyrosine kinase activity, protein kinase activity.

KEGG enrichment analysis identified 103 KEGG pathways (Figure 2B). The top 10 KEGG pathways include: Pathways in cancer, Lipid and atherosclerosis, Efferocytosis, Coronavirus disease-COVID-19, IL-17 signaling pathway, Endocrine resistance, MAPK signaling pathway, Prolactin signaling pathway, Non-alcoholic fatty liver disease, Colorectal cancer. The results suggest a potential association between MGD’s anti-AD effect and the MAPK signaling pathway.

### 3.7 Drug-component-target-pathway interaction network

The potential active components, their targets, and related pathways of MGD were imported into Cytoscape 3.9.1 to construct a drug-component-target-pathway network (Figure 2C). The network consists of 99 nodes and 724 edges. Hexagonal nodes on the left represent potential active components of Modified Gandou Decoction; circular nodes in the middle represent potential targets of Modified Gandou Decoction for AD treatment; diamond-shaped nodes on the right represent the top 10 pathways by KEGG enrichment analysis P-value (Figure 2C).

### 3.8 Molecular docking

**FIGURE 3:**
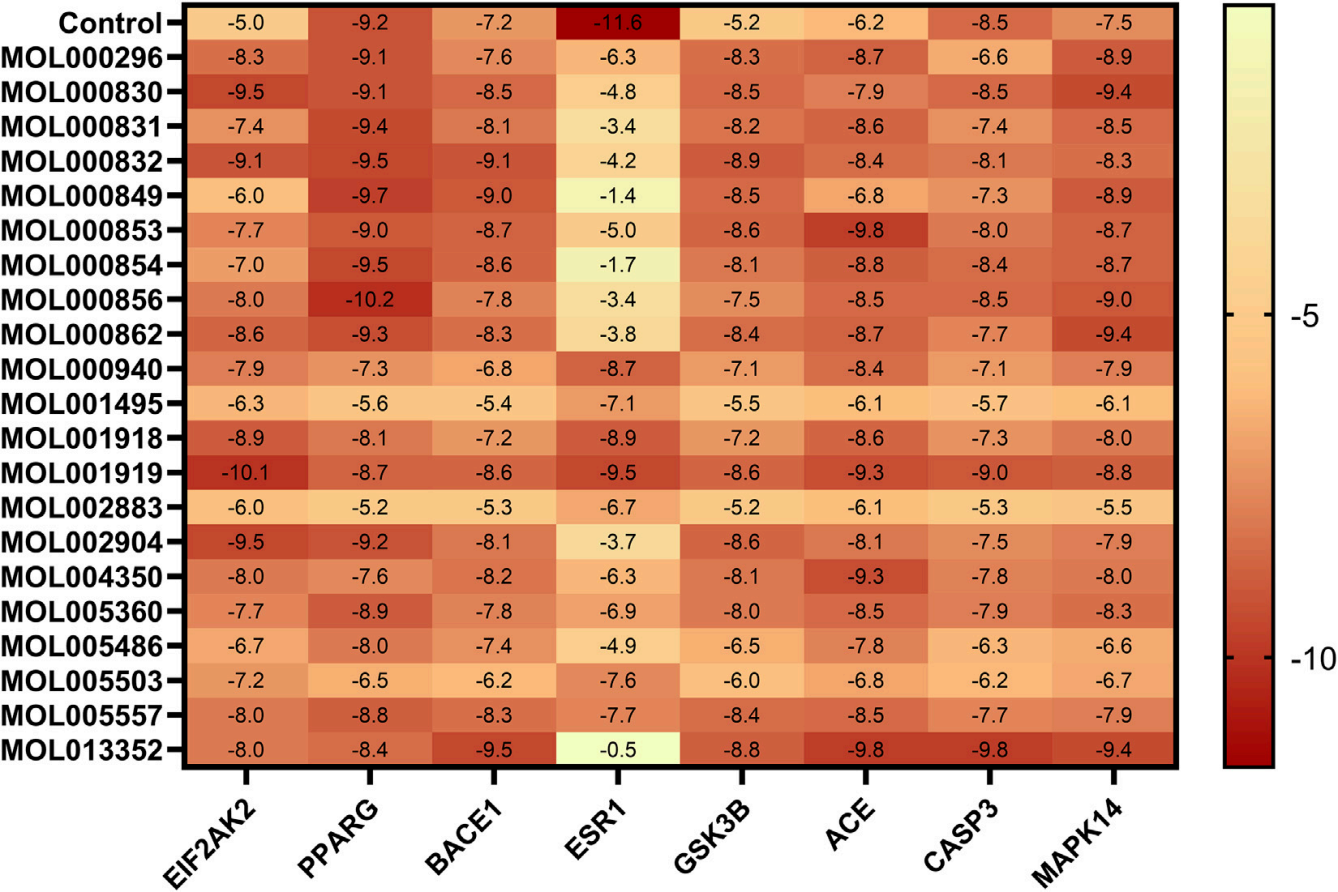
Heat map of molecular docking binding energy. Binding energy (kcal/mol) of 26 potential active molecules with 8 core targets from molecular docking, compared with positive control molecules of each core target. Darker colors indicate lower binding energy.

The 21 potential active molecules of MGD were docked with the 8 core target proteins of AD. Core target protein structure data were obtained from PDB: 1GFW (CASP3), 7MSA (ESR1), 1Q3W (GSK3B), 1UZF (ACE), 2RG6 (MAPK14), 2OHP (BACE1), 6D3K (EIF2AK2). After docking, the binding energy results were plotted as a heat map (Figure 3). Darker colors indicate lower binding energy between proteins and small molecules, and more stable binding. Studies have reported that a binding free energy <−5.00 kcal/mol indicates good binding affinity, and <−7.00 kcal/mol indicates extremely strong affinity (Shah et al., 2025). Molecular docking results showed that except for ESR1 with MOL000830, MOL000831, MOL000832, MOL000849, MOL000853, MOL000854, MOL000856, MOL000862, MOL002904, MOL005486, and MOL013352, the binding energy of other MGD potential active molecules with other core target proteins was less than −5.00 kcal/mol; the lowest binding energy between PPARG and MOL000856 was −10.2 kcal/mol, and that between EIF2AK2 and MOL001919 was −10.1 kcal/mol.

**FIGURE 4:**
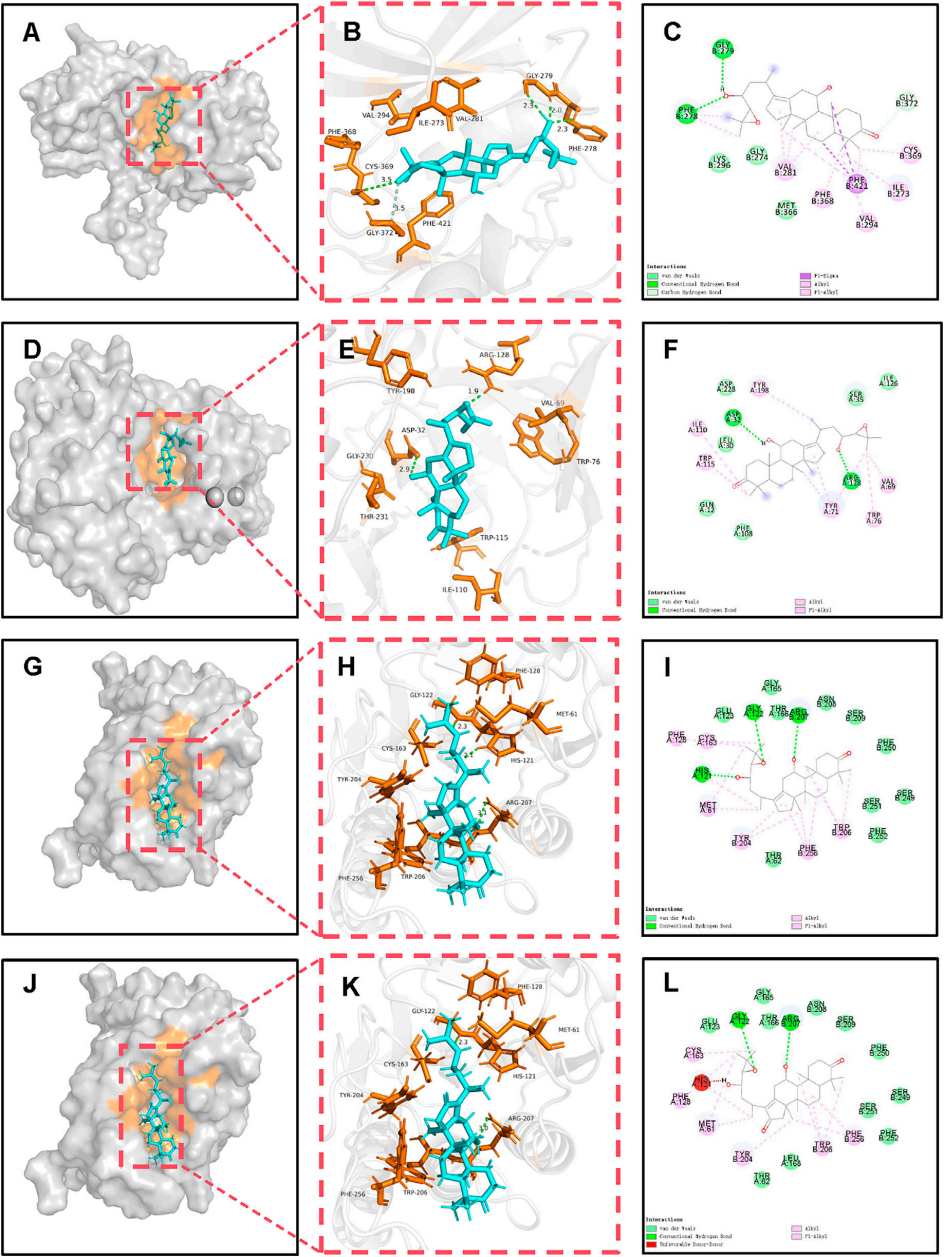
Molecular docking and binding analysis of MGD potential active molecules with BACE1, EIF2AK2, and CASP3. **(A)** Spatial structure of MOL000830-BACE1. **(B)** 3D binding of MOL000830-BACE1. **(C)** 2D binding of MOL000830-BACE1. **(D)** Spatial structure of MOL000830-EIF2AK2. **(E)** 3D binding of MOL000830-EIF2AK2. **(F)** 2D binding of MOL000830-EIF2AK2. **(G)** Spatial structure of MOL000830-CASP3. **(H)** 3D binding of MOL000830-CASP3. **(I)** 2D binding of MOL000830-CASP3. **(J)** Spatial structure of MOL000854-CASP3. **(K)** 3D binding of MOL000854-CASP3. **(L)** 2D binding of MOL000854-CASP3.

The majority of the potential active components in MGD demonstrated binding affinity with most of the core targets, indicating that the anti-AD effects of MGD are likely mediated through multi-component, multi-target, and multi-pathway mechanisms (Figure 3). The binding modes of MGD’s potential active components and positive controls with core target proteins were analyzed, and docking results with low binding energy and similar binding modes to positive controls were selected for display (Supplementary Table S2; Figure 4).

In terms of binding energy and similarity of binding to positive controls, potential active molecules showed good similarity to positive controls in binding to target proteins, and some even had advantages. The binding energy of MOL000830 with EIF2AK2 (−9.5 kcal/mol) was lower than that of its positive control 2-AP (a known EIF2AK2 inhibitor, −5.0 kcal/mol); 2-AP formed only 3 bonds with EIF2AK2 (2 hydrogen bonds and 1 hydrophobic bond), while MOL000830 formed a total of 9 bonds with EIF2AK2; they shared 2 identical bonds, including CYS369 and VAL294 (Supplementary Table S2). The binding energy of MOL000830 with BACE1 (−8.5 kcal/mol) was lower than that of its positive control 6IP (−7.2 kcal/mol); 6IP formed only 3 hydrogen bonds with BACE1, while MOL000830 formed a total of 8 bonds with BACE1; they shared only 1 identical bond, i.e., ASP32 (Supplementary Table S2). MOL000830 (Alisol B) and MOL000854 (Alisol C) showed good binding to target protein CASP3, with binding energies of −8.5 kcal/mol and −8.4 kcal/mol, respectively; the binding energy of MOL000830 with CASP3 was the same as that of the positive control, and the binding energy of MOL00854 with CASP3 was slightly higher than that of its positive control (−8.5 kcal/mol); MOL000830 and MOL000854 shared the following identical bonds with the positive control MSI in binding to CASP3: ARG207, GLY-122, TRY-204, and PHE-256, showing good similarity. The binding energy of MOL000832 with target protein MAPK14 was −8.3 kcal/mol, lower than that of the positive control (−7.2 kcal/mol); they shared the same hydrogen bond binding site ASP-168, and the same π-alkyl interaction sites VAL-38, ALA-51, and ILE-84.

The above potential active molecules of MGD, with binding energy mostly lower than that of positive controls and similar binding modes, may exert anti-AD effects by competitively inhibiting related target proteins similarly to positive controls.

### 3.9 Molecular dynamics (MD) simulation

Molecular docking revealed that MOL000830 exhibited excellent binding affinity with multiple core targets (EIF2AK2, BACE1, and CASP3), with binding energies of −9.5, −8.5, and −8.5 kcal/mol, respectively. Given its low binding energy and docking interactions comparable to the positive controls (Supplementary Table S2; Figures 3, 4), we further investigated the binding stability of MOL000830 with these targets using 50-nanosecond all-atom molecular dynamics simulations. The following parameters during the dynamics process were analyzed: root mean square deviation (RMSD); root mean square fluctuation (RMSF); radius of gyration (Rg); number of hydrogen bonds (H-bonds); Gibbs energy landscapes; and calculation of MM/PBSA binding free energy during dynamics.

#### 3.9.1 MD simulation of MOL000830-EIF2AK2 complex

The MD simulation time was 50ns. The RMSD curve of EIF2AK2 after binding to MOL000830 (red line) was lower than that before binding (black line), and the vibration range of the EIF2AK2 complex molecular system after binding to MOL000830 was smaller, indicating that the complex showed a more stable trend after binding (Supplementary Figure S2A). Similarly, in the 50ns dynamics simulation, the RMSF of the protein polypeptide backbone atoms in the MOL000830-EIF2AK2 complex (red line) was lower than that of the free EIF2AK2 protein (black line), indicating that the protein molecular structure was more stable in each part after binding to MOL000830 (Supplementary Figure S2B). The radius of gyration (Rg) of the MOL000830-EIF2AK2 complex stabilized at 3.5nm–3.6 nm after 25ns of dynamics simulation, indicating that the complex stabilized 25ns after formation (Supplementary Figure S2C). Hydrogen bonds are the main force for binding between small drug molecules and proteins. Within 0–50ns of dynamics simulation, the number of hydrogen bonds in the interaction of the MOL000830-EIF2AK2 complex stabilized at 2-3 (Supplementary Figure S2D). In addition, the Gibbs free energy landscape of the MOL000830-EIF2AK2 complex showed that when the Rg value was 3.48–3.65 nm and RMSD was 0.9–1.29nm, the MOL000830-EIF2AK2 complex had lower Gibbs free energy, i.e., a more stable conformation (Supplementary Figure S2E). The average binding free energy of MOL000830-EIF2AK2 in the last 5ns calculated by the MM-PBSA method was −38.419 kcal/mol, and the main contributors to the binding free energy were amino acid residues of EIF2AK2: CYS-369, ILE-273, GLN-376, PHE-421, PHE-368, etc. (Supplementary Figure S2F).

MOL000830 and EIF2AK2 can form a stable complex; therefore, MOL000830 can not only occupy the binding site of EIF2AK2 spatially but also maintain stable binding over time, continuously preventing the binding of natural substrates to the target protein, thereby inhibiting the activity of the target protein. Moreover, MOL000830 and 2-Aminopurine (2-AP, a known inhibitor of EIF2AK2) share the same binding sites on EIF2AK2: CYS-369, GLN-367, and VAL-281, indicating that MOL000830 can inhibit EIF2AK2 through the same competitive inhibition, exerting anti-AD effects.

#### 3.9.2 MD simulation of MOL000830-BACE1 complex

The RMSD curves of BACE1 (black line) and the MOL000830-BACE1 complex (red line) remained relatively stable within 0–50 ns, with an average RMSD value of BACE1 of approximately 0.21 nm and that of the MOL000830-BACE1 complex of approximately 0.16 nm, indicating that the MOL000830-BACE1 complex molecule was more stable after binding (Supplementary Figure S3A). The RMSF value of the complex system decreased after binding to MOL000830, indicating that MOL000830 can form a stable complex system with BACE1 and has a stabilizing effect on the protein (Supplementary Figure S3B). Supplementary Figure S3C shows that the Rg value of the MOL000830-BACE1 complex stabilized at 2.075 nm–2.1 nm within 0–50 ns, indicating that MOL000830 binds tightly to BACE1. Within 0–50 ns, the number of hydrogen bonds in the MOL000830-BACE1 complex was 0–5 (Supplementary Figure S3D). The Gibbs free energy landscape of the MOL000830-BACE1 complex showed that there was only one main free energy basin in the entire free energy landscape, indicating that the complex can reach a metastable conformation by crossing only one energy barrier, and the basin was broad and deep, indicating that the interaction between BACE1 and MOL000830 was strong and stable during the entire simulation process; when the Rg value was 2.08–2.1 nm and RMSD was 0.15 nm, the MOL000830-BACE1 complex had lower Gibbs free energy, i.e., a more stable conformation (Supplementary Figure S3E). The average binding free energy of MOL000830-BACE1 in the last 5ns calculated by the MM-PBSA method was −21.546 kcal/mol, and the main contributing residues were ILE-118, TYR-71, ILE-110, TRP-76, and VAL-69 (Supplementary Figure S3F).

Molecular dynamics results showed that MOL000830 can form a stable complex with BACE1 with tight binding. In the simulation, MOL000830 and 6-[2-(1H-INDOL-6-YL) ETHYL] PYRIDIN-2-AMINE (an inhibitor of BACE1) shared the same binding sites ASP-228 and PHE-108, indicating that MOL000830 may inhibit BACE1 through the same competitive inhibition.

#### 3.9.3 MD simulation of MOL000830-CASP3 complex

Supplementary Figure S4A shows that the RMSD of CASP3 stabilized at 0.15 nm–0.3 nm (black line), and the MOL000830-CASP3 complex stabilized at 0.10 nm–0.28 nm (red line); the MOL000830-CASP3 complex was more stable within 0–50 ns. The RMSF value of the complex system slightly decreased after binding to MOL000830, indicating that MOL000830 can form a relatively stable complex with CASP3 (Supplementary Figure S4B). The Rg value of the MOL000830-CASP3 complex stabilized at 1.78 nm–1.83 nm within 0–50 ns, indicating that MOL000830 binds tightly to BACE1 (Supplementary Figure S4C). Within 0–50 ns, the number of hydrogen bonds in the MOL000830-CASP3 complex was 1–3 (Supplementary Figure S4D). The Gibbs free energy landscape of the MOL000830-CASP3 complex showed that there was only one main free energy basin in the entire free energy landscape, indicating that the complex can reach a metastable conformation by crossing only one energy barrier; the basin was broad and deep, indicating that the interaction between CASP3 and MOL000830 was strong and stable during the entire simulation process; when the Rg value was 1.75–1.80 nm and RMSD was 0.15–0.24 nm, the MOL000830-CASP3 complex had lower Gibbs free energy and a more stable conformation (Supplementary Figure S4E). The average binding free energy of MOL000830-CASP3 in the last 5 ns calculated by the MM-PBSA method was −11.176 kcal/mol, and the main contributing residues were PHE-256, TYR-204, TRP-206, THR-166, and LEU-168 (Supplementary Figure S4F).

MD Simulation of MOL000830-CASP3 Complex results showed that MOL000830 can form a stable complex with CASP3 with tight binding. In the simulation, MOL000830 and 1-METHYL-5-(2-PHENOXYMETHYL-PYRROLIDINE-1-SULFONYL)-1H-INDOLE-2,3-DIONE (an inhibitor of CASP3) shared the same binding sites TYR-204, TRP-206, and PHE-256, indicating that MOL000830 can inhibit CASP3 through the same competitive inhibition, exerting anti-AD effects.

### 3.10 CCK-8 assay for cell viability

**FIGURE 5:**
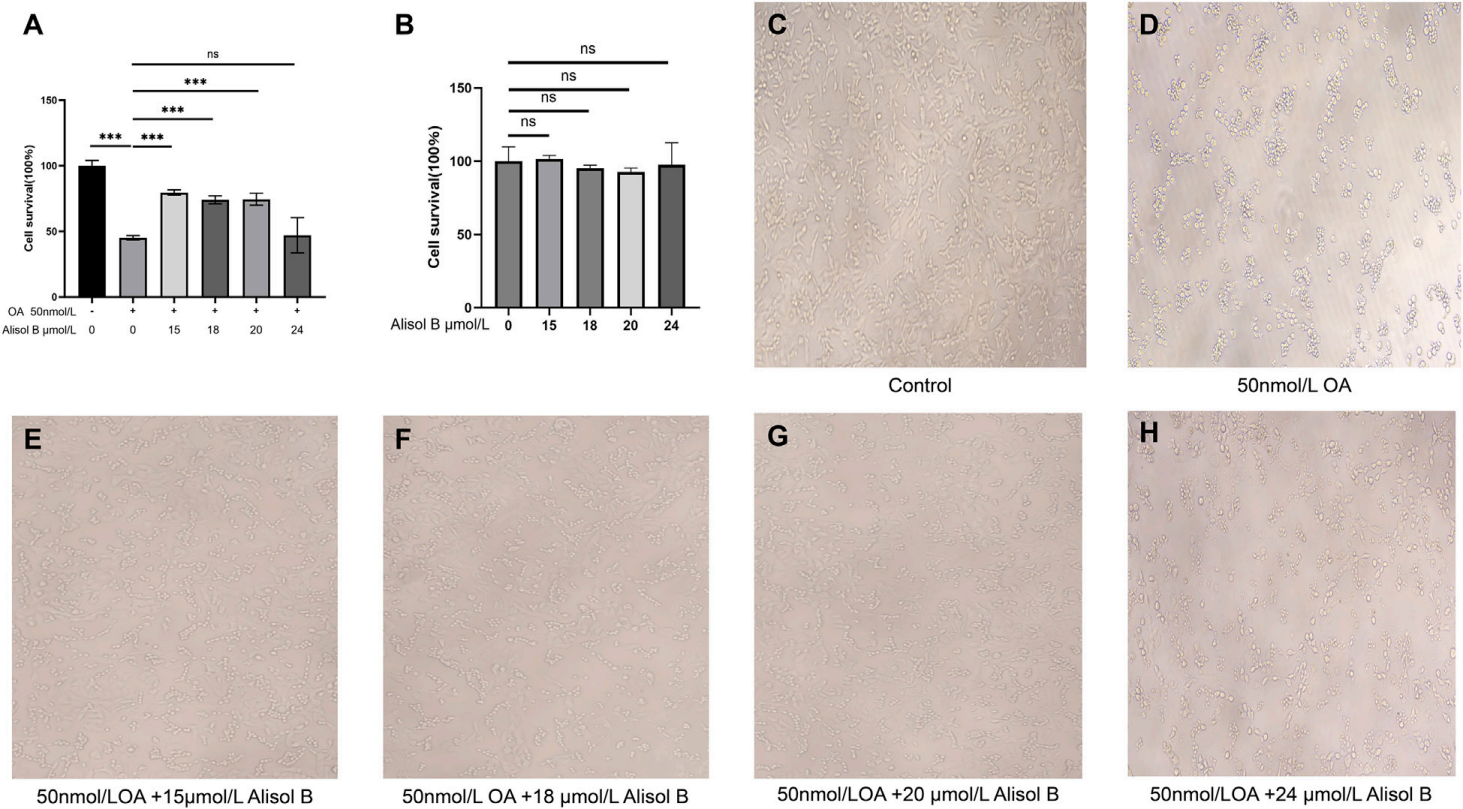
Alisol B reverses OA-induced reduction in SHSY5Y cell viability. **(A)** Cell viability measured by CCK-8 assay: Control group, OA group, 15 μmol/L Alisol B + 50 nmol/L OA group, 18 μmol/L Alisol B + 50 nmol/L OA group, 20 μmol/L Alisol B + 50 nmol/L OA group, 24 μmol/L Alisol B + 50 nmol/L OA group. **(B)** Control group, 15 μmol/L Alisol B group, 18 μmol/L Alisol B group, 20 μmol/L Alisol B group, 24 μmol/L Alisol B group. **(C)** Microscopic observation of cell status (magnification ×40) in the Control group. **(D)** OA group. **(E)** 15 μmol/L Alisol B + 50 nmol/L OA group. **(F)** 18 μmol/L Alisol B + 50 nmol/L OA group. **(G)** 20 μmol/L Alisol B + 50 nmol/L OA group. **(H)** 24 μmol/L Alisol B + 50 nmol/L OA group.

Okadaic acid (OA) can induce excessive phosphorylation of the cytoskeleton, thereby causing nerve cell damage similar to that caused by Aβ-induced Tau protein phosphorylation, which reduces microtubule stability (Shen et al., 2018). In this study, OA (concentration 50 nmol/L) was used to inhibit the viability of SH-SY5Y human neuroblastoma cells to establish a neural cell model of AD. Then, different concentrations of MOL000830 (Alisol B) molecules were added to protect SH-SY5Y cells and enhance their viability. The CCK-8 assay revealed that compared with the control group, treatment of SH-SY5Y cells with 50 nmol/L OA for 6 h significantly reduced the cell survival rate to 45.12% of that of untreated cells (p < 0.001) (Figure 5A). After OA treatment, cells were simultaneously treated with different concentrations of Alisol B. The results showed that 15 μmol/L, 18 μmol/L, and 20 μmol/L Alisol B significantly increased the survival rate of OA-treated SH-SY5Y cells (p < 0.001), with survival rates of 79.55%, 74.38%, and 74.48%, respectively; compared with the OA-treated group alone, the survival rates were 1.76, 1.65, and 1.65 times that of the OA-treated group alone, but still did not reach the cell viability of the blank group (p < 0.001) (Figure 5A). Treatment with 24 μmol/L Alisol B after OA treatment resulted in a cell survival rate of 46.98%, with no effect on improving cell survival rate (p < 0.001) (Figure 5A). Notably, the control groups treated with Alisol B at concentrations corresponding to those in the experimental groups exhibited no significant effect on cell viability (Figure 5B).

Microscopic observation of cells in each group of the CCK-8 experiment at ×40 magnification yielded the same results (Figures 5C–H). Therefore, Alisol B at 15 μmol/L significantly rescued OA-induced cell damage, while 24 μM showed no protective effect.

## 4 Discussion

MGD is a classic TCM compound consisting of 9 Chinese herbs: *Rhubarb*, *Coptis chinensis*, *Curcuma zedoaria*, *Curcuma longa*, *Houttuynia cordata*, *Alisma orientale*, *Panax notoginseng*, *Paeonia lactiflora*, and *Cornus officinalis*. MGD has been demonstrated to ameliorate neuronal damage in the brains of Wilson’s disease model mice induced by copper overload through two distinct mechanisms: by regulating the Cyt c/caspase signaling pathway through facilitating the excretion of excess copper and suppressing the expression of Cyt c, Caspase-9, and caspase-3 in neurons; and concurrently by downregulating the mRNA and protein expression levels of acid sphingomyelinase (ASM), ceramide (Cer), and p38 MAPK in the ceramide signaling pathway (Xu et al., 2017b). In this study, 21 potential active components were identified from MGD according to the criteria of OB, DL, Lipinski’s five rules, blood-brain barrier permeability, and no hepatotoxicity; they are mainly derived from *Coptis chinensis*, *Curcuma zedoaria*, *Houttuynia cordata*, *Alisma orientale*, *Paeonia lactiflora*, and *Cornus officinalis* (Supplementary Table S1).

Further intersection analysis of drug targets and AD-related targets identified 68 potential targets for MGD in AD treatment. GO function analysis and KEGG pathway analysis showed that the potential active components of MGD intervene in AD-related pathways by acting on disease targets, including pathways in cancer, lipid and atherosclerosis pathways, IL-17 signaling pathway, MAPK signaling pathway, etc. (Figures 2A,B). Machine learning focused on core targets, and finally, 8 core targets were screened: EIF2AK2, PPARG, BACE1, ESR1, GSK3B, ACE, CASP3, and MAPK14 (Figure 1G). The roles of these targets in AD pathogenesis include: EIF2AK2, involved in cellular stress response and protein synthesis regulation (Feng et al., 2025), BACE1, closely related to Aβ peptide production (Kapasi et al., 2021), and CASP3, playing a key role in cell apoptosis (Feng et al., 2025). Differential expression analysis of core targets in hippocampal tissues of AD patients and normal tissues showed that except for PPARG, other core targets were significantly upregulated in hippocampal tissues of AD patients (Supplementary Figure S1). This finding further validates the importance of the core targets in AD.

Molecular docking results showed that the binding of MGD’s potential active molecules to AD core targets has the following characteristics: most of the 21 potential active molecules have strong binding to core target proteins, with binding energy <−5 kcal/mol; almost all 21 potential active molecules can bind to the 8 core proteins of AD; each core protein can bind to multiple potential active molecules (Figure 3). This fully indicates that MGD’s therapeutic effect on AD has a synergistic effect through multiple components, targets, and pathways.

Interestingly, except for poor binding to ESR1, Alisol B (MOL000830) has good binding to the other 7 core proteins, mainly reflected in low binding energy and similar binding to corresponding positive controls (Supplementary Table S2; Figures 3, 4). This characteristic of a small molecule binding to multiple target proteins suggests that it may simultaneously intervene in β-amyloid production and cellular stress response pathways, producing a synergistic inhibitory effect. Compared with drug molecules acting on a single target protein, Alisol B has greater potential for drug development. Further molecular dynamics simulations confirmed that Alisol B can not only bind to EIF2AK2, BACE1, and CASP3 spatially to occupy the binding site and prevent the binding of their natural substrates but also maintain stable binding over time, continuously preventing natural substrates from binding to target proteins, thereby inhibiting the activity of target proteins.

**FIGURE 6:**
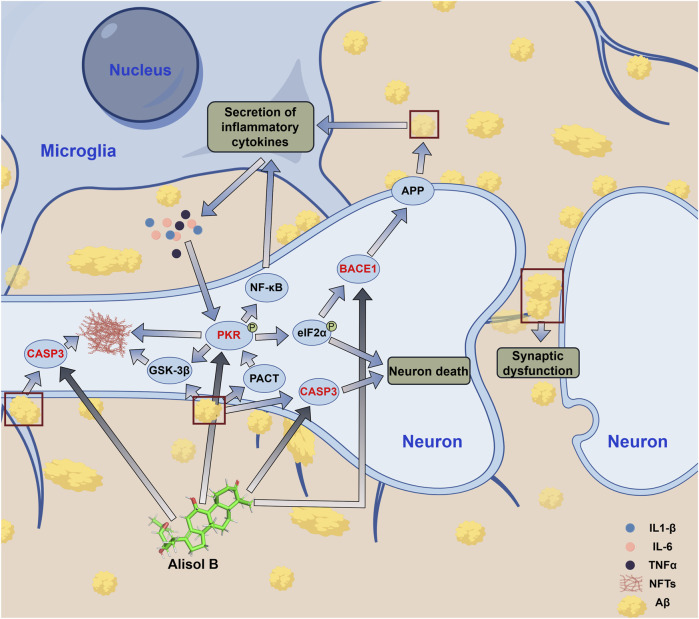
Related signaling pathways of Alisol B inhibiting AD.

BACE1 belongs to the aspartic protease family, and its main function is to cleave amyloid precursor protein (APP) to cause Aβ production and accumulation (Hampel et al., 2021). APP cleavage has two pathways: the non-amyloidogenic pathway mediated by α-secretase, and the amyloidogenic pathway mediated by β-secretase (BACE1) and γ-secretase (Chen et al., 2016). BACE1 is a key enzyme for Aβ production; therefore, inhibiting BACE1 can directly reduce Aβ production, thereby delaying AD progression (Pan et al., 2024). Alisol B can bind to BACE1; therefore, Alisol B can directly inhibit Aβ production (Supplementary Figure S3; Figure 6).

EIF2AK2, also known as double-stranded RNA-activated protein kinase (PKR), is one of the human innate immune interferon-stimulating factors and a pro-inflammatory cytokine (Mañucat-Tan et al., 2019). EIF2AK2 is involved in various physiological processes, including antiviral defense, protein translation regulation, cell apoptosis and proliferation, innate immunity, and inflammatory response. Studies have shown that aggregated Aβ can induce upregulation of brain tissue PKR activator (PACT) expression and activate EIF2AK2 (Ramasamy et al., 2025); activated EIF2AK2 phosphorylates eukaryotic protein translation initiation factor 2α (eIF2α), thereby shutting down eIF2α-dependent protein translation initiation. This shutdown of protein translation initiation instead promotes eIF2α-independent but mRNA upstream ORFs (uORFs)-dependent translation initiation of BACE1, thereby promoting BACE1 expression and further increasing Aβ production, forming a cycle that promotes Aβ production and accumulation (Mihailovich et al., 2007; Moradi Majd and Mayeli, 2020). In addition to promoting Aβ production, EIF2AK2 activation can promote the production and release of inflammatory cytokines such as IL6, IL1-β, and TNFα through the NF-κB pathway, causing cellular inflammatory responses and nerve cell apoptosis (Hugon et al., 2017). Moreover, EIF2AK2 activation can promote Tau protein phosphorylation, reduce the stability of neuronal microtubules, and cause them to entangle with each other, forming neurofibrillary tangles (NFTs); EIF2AK2 activation can also further promote NFTs production by activating GSK-3β (Otero-Garcia et al., 2022). EIF2AK2 activation causes continuous Aβ accumulation, leading to neuronal inflammatory responses and cell apoptosis; in addition, Tau protein phosphorylation seriously affects the stability of neuronal microtubule cytoskeleton, causing axonal signal transduction disorders and leading to memory and cognitive decline (Figure 6). Alisol B binding to EIF2AK2 can inhibit its activation, effectively inhibiting Aβ accumulation and the resulting neuroinflammation; interrupting the cycle of EIF2AK2 activation, activating BACE1 and further causing Aβ accumulation; and simultaneously inhibiting Tau phosphorylation, preventing the depolymerization of neuronal microtubule cytoskeleton and the formation of NFTs, effectively controlling AD progression (Supplementary Figure S2; Figures 4, 6). Therefore, EIF2AK2 plays a key role in AD occurrence and progression, and inhibiting EIF2AK2 is expected to effectively control AD progression.

CASP3 is caspase-3, belonging to the cysteine protease family, mainly involved in the cell apoptosis process; it can directly mediate β-amyloid (Aβ)-induced neuronal apoptosis, participate in abnormal processing of Tau protein and NFTs formation, and is also an important protein in the MAPK signaling pathway (Floden et al., 2005; Yoshida and Goedert, 2006; Wang et al., 2014; Shao et al., 2018). The results of this study showed that both Alisol B and Alisol C can stably bind to CASP3 (Figures 3, 4). Therefore, Alisol B and alisol C binding to CASP3 can inhibit its activity and neuronal apoptosis, exerting anti-AD effects. A previous study demonstrated that MGD inhibits the expression of Caspase-3 in neurons. This agreement with the predictions of our current study lends credence to the reliability of our molecular docking and molecular dynamics simulation analyses (Xu et al., 2017a).

Feng et al. (2025) synthesized a circular RNA that can bind to the double-stranded RNA-binding domain of EIF2AK2, prevent EIF2AK2 dimerization, and inhibit EIF2AK2 activation, successfully alleviating neuroinflammation and AD symptoms in AD model mice. The EIF2AK2 inhibitor C16 can reduce cognitive memory impairment, neurodegeneration, neuroinflammation, and Aβ accumulation in the brain (Ingrand et al., 2007). Studies have reported that Alisol B has anti-tumor effects, suggesting that its inability to enhance cell survival rate at 24 μmol/L may be related to its anti-tumor effect (Xu et al., 2018). This study used OA to act on SH-SY5Y human neuroblastoma cells to establish an AD cell model; CCK8 cell experiment results showed that Alisol B can reduce OA-induced decrease in AD cell viability (Figure 5A). These research results support that the potential active component Alisol B in MGD can target and inhibit EIF2AK2, BACE1, and CASP3, thereby having potential applications in AD treatment.

Network pharmacology analysis showed that Alisol B is a potential active molecule derived from the Chinese herb *Alisma orientale*. Chen et al. (Chen et al., 2024) reported through UHPLC-Q-Orbitrap HRMS that Alisol B and its concentration can be identified in the serum of mice fed with Alisma Decoction, indicating that Alisol B is stable during Chinese herb decoction and can be well absorbed into the blood through the mouse intestine. ADMET analysis showed that Alisol B can cross the blood-brain barrier (Supplementary Table S1). This indicates that Alisol B has the characteristics of an AD therapeutic drug.

Although this study investigated the anti-AD potential of MGD through multi-faceted computational simulations and preliminary experiments, several limitations should be acknowledged. Firstly, the effects of Alisol B were only verified through *in vitro* cell experiments, lacking *in vivo* validation using AD animal models (such as behavioral assessment and brain tissue pathological examination), resulting in insufficient support for clinical translation. Secondly, the synergistic or antagonistic effects among the 21 potential active ingredients in MGD were not explored, failing to fully reflect the “multi-component - multi-target” characteristics of traditional Chinese medicine formulas. Thirdly, only SH-SY5Y cells were used in the *in vitro* experiments, and only cell viability was detected, leading to a single model and detection dimension. Fourthly, no pharmacokinetic studies or safety assessments of Alisol B were conducted, limiting its reference value for drug development. Finally, the regulatory network among core targets was not adequately analyzed, and the depth of mechanism elucidation was insufficient. In the future, *in vivo* validation could be improved by constructing AD animal models. The synergistic mechanisms of multiple components could be analyzed using techniques such as UPLC-Q-TOF/MS. The experimental models and detection systems could be optimized. Pharmacokinetic and safety assessments could be carried out. Research on the regulatory network of targets could be deepened to improve the anti-AD mechanism of MGD and promote clinical translation.

In conclusion, this study screened 21 potential active molecules of MGD for AD treatment and their 8 core targets in AD through network pharmacology and artificial intelligence analysis. Further differential expression analysis of core target genes confirmed the effectiveness of core target upregulation for potential active molecule binding. Molecular docking results showed that most of the 21 potential active molecules have strong binding to the 8 core target proteins, indicating that MGD has a synergistic effect through multiple components, targets, and pathways in alleviating cognitive decline in AD. Molecular dynamics results confirmed that Alisol B can stably bind to BACE1, EIF2AK2, and CASP3, inhibiting Aβ production and accumulation, Tau protein phosphorylation and NFTs formation, neuroinflammation, and inflammation-induced neuronal apoptosis, thereby alleviating AD occurrence and progression. CCK8 cell experiment results further confirmed the protective effect of Alisol B on OA-induced damage in SH-SY5Y human neuroblastoma cells. This study can provide a basis for the use of MGD in alleviating cognitive decline in AD and useful insights for the development of AD therapeutic drugs, but its specific mechanism requires further research verification.

## Data Availability

The datasets presented in this study can be found in online repositories. The names of the repository/repositories and accession number(s) can be found in the article/Supplementary Material.
