# Supplementary material for: Screening and experimental validation of modified Gandou Decoction-targeted inhibitors for alleviating AD components via network pharmacology, machine learning, and molecular dynamics simulation
Source: Front Pharmacol. 2025 Oct 31;16:1685866. doi: 10.3389/fphar.2025.1685866 (PMC12615429; doi:10.3389/fphar.2025.1685866)
Supplement: Supplementary file 1 [file DataSheet1.pdf]

Supplementary File 1

Supplementary Table 1. Twenty-One Potential Active Components of Modified Gandou Decoction

MOL ID	Description	OB (%)	DL	BBB	Hepatotoxicity
MOL000296	Hederagenin	36.91	0.75	(+)	(-)
MOL000830	Alisol B	34.47	0.82	(+)	(-)
MOL000831	Alisol B monoacetate	35.58	0.81	(+)	(-)
MOL000832	alisol,b,23-acetate	32.52	0.82	(+)	(-)
MOL000849	16β-methoxyalisol B monoacetate	32.43	0.77	(+)	(-)
MOL000853	alisol B	36.76	0.82	(+)	(-)
MOL000854	alisol C	32.7	0.82	(+)	(-)
MOL000856	alisol C monoacetate	33.06	0.83	(+)	(-)
MOL000862	[(1S,3R)-1-[(2R)-3,3-dimethyloxiran-2-yl]-3-[(5R,8S,9S,10S,11S,14R)-11-hydroxy-4,4,8,10,14-pentamethyl-3-oxo-1,2,5,6,7,9,11,12,15,16-decahydrocyclopenta[a]phenanthren-17-yl]butyl] acetate	35.58	0.81	(+)	(-)
MOL000940	bisdemethoxycurcumin	77.38	0.26	(+)	(-)
MOL001495	Ethyl linolenate	46.1	0.2	(+)	(-)
MOL001918	paeoniflorgenone	87.59	0.37	(+)	(-)
MOL001919	(3S,5R,8R,9R,10S,14S)-3,17-dihydroxy-4,4,8,10,14-pentamethyl-2,3,5,6,7,9-hexahydro-1H-cyclopenta[a]phenanthrene-15,16-dione	43.56	0.53	(+)	(-)
MOL002883	Ethyl oleate (NF)	32.4	0.19	(+)	(-)
MOL002904	Berlambine	36.68	0.82	(+)	(-)
MOL004350	Ruvoside_qt	36.12	0.76	(+)	(-)
MOL005360	malkangunin	57.71	0.63	(+)	(-)
MOL005486	3,4-Dehydrolycopen-16-al	46.64	0.49	(+)	(-)

MOL ID	Description	OB (%)	DL	BBB	Hepatotoxicity
MOL005503	Cornudentanone	39.66	0.33	(+)	(-)
MOL005557	lanosta-8,24-dien-3-ol,3-acetate	44.3	0.82	(+)	(-)
MOL013352	Obacunone	43.29	0.77	(+)	(-)

Supplementary Table 2. Analysis of binding interactions between small molecules and target proteins.

Compound	Binding energy(k cal/mol)	Conventional Hydrogen Bond	Carbon Hydrogen Bond	Alkyl/Pi-Alkyl	Van der Waals
(2-AP)-EIF2AK2 (Positive control)	-5.0	GLN-367<br>CYS-369	-	VAL-294	VAL-281<br>VAL-321
MOL000830-EIF2AK2	-9.5	CYS-369<br>PHE-278<br>GLY-279	GLY-372	ILE-273<br>VAL-281<br>VAL-294<br>PHE-368	GLY-274<br>LYS-296<br>MET-366
6IP-BACE1 (Positive control)	-7.2	ASP-32<br>LYS-107<br>ASP-228	-	-	GLY-34<br>GLN-73<br>GLY-74<br>GLY-230<br>PHE-108
MOL000830-BACE1	-8.5	ASP-32<br>ARG-128	-	VAL-69<br>TYR-71<br>TRP-76<br>ILE-110<br>TRP-115<br>TYR-198	GLN-12<br>LEU-30<br>SER-35<br>PHE-108<br>ILE-126<br>ASP-228
MSI-CASP3 (Positive control)	-8.5	GLY-122	SER-205<br>HIS-121<br>GLY-122	TYR-204<br>TRP-206<br>ARG-207<br>PHE-256	-
MOL000830-CASP3	-8.5	ARG-207<br>HIS-121<br>GLY-122	-	TYR-61<br>HIS-128<br>CYS-163<br>TYR-204<br>TRP-206<br>PHE-256	-

Compound	Binding energy(kcal/mol)	Conventional Hydrogen Bond	Carbon Hydrogen Bond	Alkyl/Pi-Alkyl	Van der Waals
MOL000854-CASP3	8.4	ARG-207<br>GLY-122	-	MET-61<br>PHE-128<br>CYS-163<br>TRP-204<br>TRP-206<br>PHE-256	-
Isorhamnetin-MAPK14<br>(Positive control)	-7.5	ASP-168	-	VAL-38<br>ALA-51<br>LEU-75<br>ILE-84<br>LEU-167	-
MOL000832-MAPK14	-8.3	GLU-71<br>THR-106<br>MET-109<br>ASP-168	LEU-108<br>LEU-167<br>PHE-169	VAL-30<br>ALA-51<br>ILE-84	-

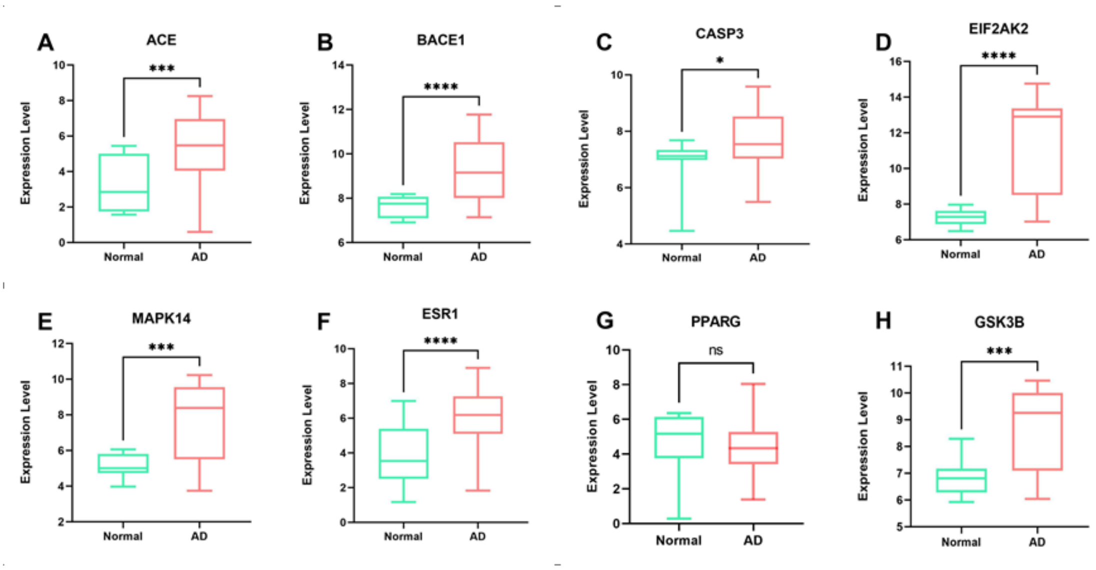

Supplementary Figure 1. MRNA expression analysis of core genes.

A. mRNA expression analysis of ACE. B. mRNA expression analysis of BACE1. C. mRNA expression analysis of CASP3. D. mRNA expression analysis of EIF2AK2. E. mRNA expression analysis of MAPK14. F. mRNA expression analysis of ESR1. G. mRNA expression analysis of PPARG. H. mRNA expression analysis of GSK3B.

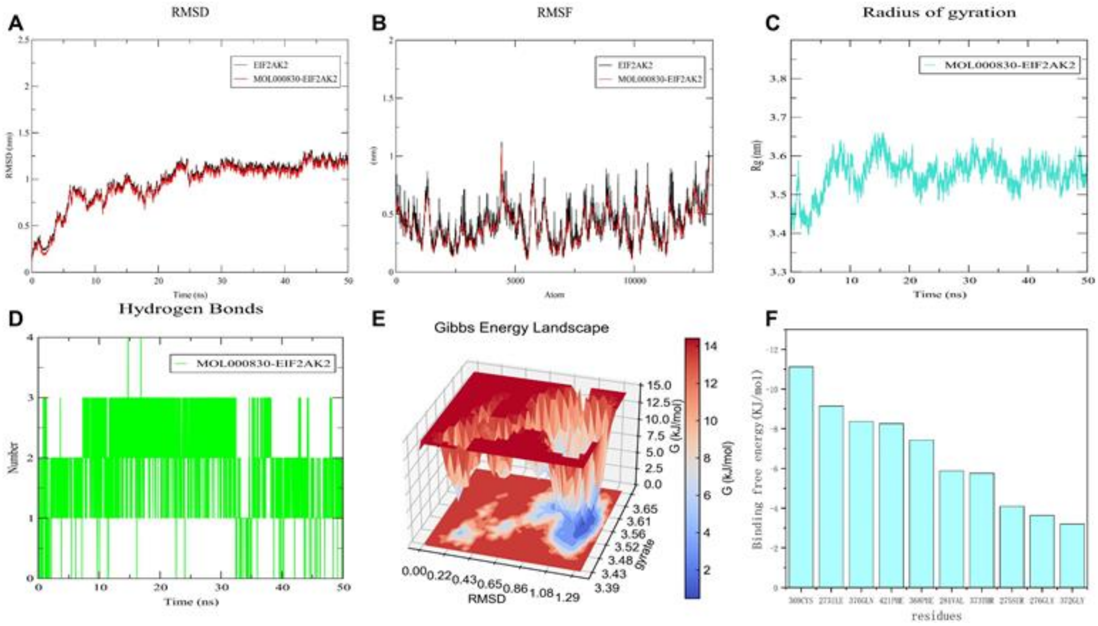

Supplementary Figure 2. Molecular dynamics simulation analysis of MOL000830-EIF2AK2.

A. RMSD of MOL000830-EIF2AK2. B. RMSF of MOL000830-EIF2AK2. C. Rg of MOL000830-EIF2AK2. D. Number of hydrogen bonds in MOL000830-EIF2AK2. E. Gibbs energy landscape of MOL000830-EIF2AK2. F. Free energy contribution of protein residues in MOL000830-EIF2AK2.

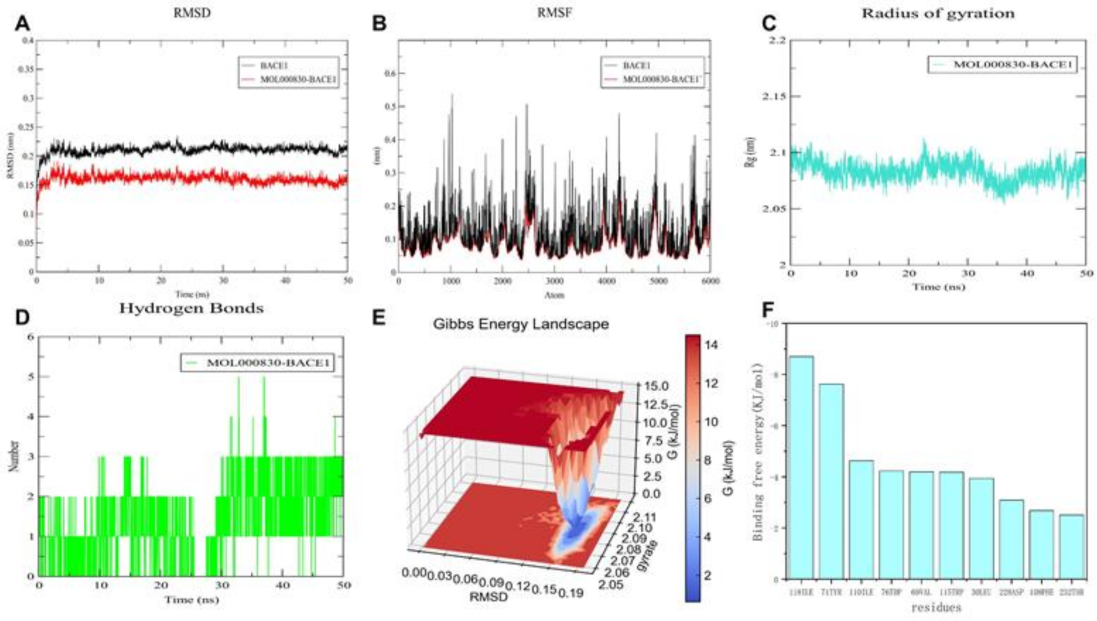

Supplementary Figure 3. Molecular dynamics simulation analysis of MOL000830-BACE1.

A. RMSD of MOL000830-BACE1. B. RMSF of MOL000830-BACE1. C. Rg of MOL000830-BACE1. D. Hydrogen bonds of MOL000830-BACE1. E. Gibbs energy landscape of MOL000830-BACE1. F. Free energy contribution of protein residues in MOL000830-BACE1.

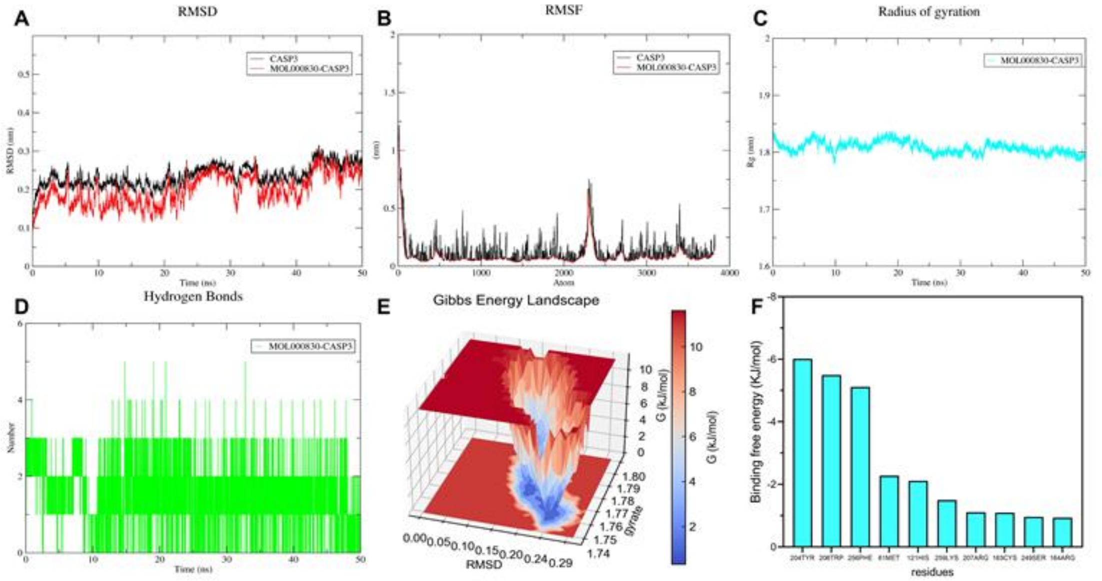

Supplementary Figure 4. Molecular dynamics simulation analysis of MOL000830-CASP3.

A. RMSD of MOL000830-CASP3. B. RMSF of MOL000830-CASP3. C. Rg of MOL000830-CASP3. D. Number of hydrogen bonds in MOL000830-CASP3. E. Gibbs energy landscape of MOL000830-CASP3. F. Free energy contribution of protein residues in MOL000830-CASP3.
